# 急性髓系白血病融合基因表达特点分析

**DOI:** 10.3760/cma.j.issn.0253-2727.2021.06.007

**Published:** 2021-06

**Authors:** 麒 闫, 亚妮 蔺, 先琪 黄, 凌志 钱, 婧婷 马, 虹 张, 龙 陈, 雪晶 陈, 营昌 秘, 昆 汝

**Affiliations:** 1 中国医学科学院、北京协和医学院血液病医院（中国医学科学院血液学研究所），实验血液学国家重点实验室，国家血液系统疾病临床医学研究中心，天津 300020 State Key Laboratory of Experimental Hematology, National Clinical Research Center for Blood Diseases, Institute of Hematology & Blood Diseases Hospital, Chinese Academy of Medical Sciences & Peking Union Medical College, Tianjin 300020, China; 2 天津见康华美医学诊断中心 300385 SINO-US Diagnostics Lab, Tianjin 300385, China

**Keywords:** 白血病，髓系，急性, 融合基因, 基因突变, 免疫表型, Leukemia, myeloid, acute, Fusion genes, Gene mutation, Immunophenotype

## Abstract

**目的:**

分析初诊急性髓系白血病（AML）患者融合基因表达情况，进一步探讨不同融合基因家族患者的免疫表型及基因突变的特点。

**方法:**

回顾性分析2016至2020年4192例初诊AML患者的多重融合基因筛查结果，并结合免疫表型分析以及高通量测序所获得的基因突变筛查结果，系统地进行差异性分析。

**结果:**

①4192例AML患者中1948例(46.47％)检出融合基因，共检出29种不同的融合基因，其阳性率呈现为“幂律分布”。②随着AML患者年龄增长，融合基因的阳性率先上升而后逐步下降。儿童患者融合基因总阳性率及MLL相关融合基因（MLL-FG）阳性率均显著高于成人患者（69.18％对44.76％，15.35％对8.36％）。③MLL-FG及NUP98相关融合基因（NUP98-FG）阳性患者伴随的基因突变均以FLT3及RAS信号通路相关基因为主。④MLL-FG及NUP98-FG阳性患者未见特异性的免疫表型特点。

**结论:**

采用多重PCR方法分析，近半数的AML患者伴有特异的融合基因表达，儿童和成人患者融合基因阳性类型构成比例存在差异。不同融合基因阳性AML患者常见的分子突变、免疫表型有一定规律。

## Abstract (en)

**Objective:**

To analyze the genetic landscape of multiple fusion genes in patients with de novo acute myeloid leukemia（AML）and investigate the characteristics of immunophenotypes and mutations.

**Methods:**

The results of multiple fusion genes from 4192 patients with de novo AML were retrospectively analyzed from 2016 to 2020. In addition, the immunophenotypical data and the mutational results from high-through put method were statistically investigated and correlated as well.

**Results:**

① Among the 52 targets, 29 different types of fusion genes were detected in 1948 patients （46.47％）with AML, which demonstrated an “exponential distribution”. ② As the age increased, the number of patients with fusion gene increased first and then decreased gradually. The total incidence rate of fusion genes and MLL rearrangment in children were significantly higher than those in adults（69.18％ *vs* 44.76％, 15.35％ *vs* 8.36％）. ③ The mutations involving FLT3 and RAS signaling pathway contributed most in patients with MLL rearrangment. ④ No specific immunophenotypic characteristics were found in AML patients with MLL or NUP98 rearrangements.

**Conclusion:**

Nearly half of AML patients were accompanied by specific fusion gene expression, the proportions of different fusion genes in pediatric and adults patients were different by multiple PCR. The gene mutations and immunophenotype of these AML patients have certain rules.

急性白血病（AL）是一组高度异质性的血液系统肿瘤，主要分为急性髓系白血病（AML）、急性淋巴细胞白血病（ALL）和系列不清的急性白血病（主要为混合表型急性白血病，MPAL）[1]–[3]，其中AML是成人最常见的AL，在全球范围内，AML发病率逐年上升[4]。

BCR-ABL融合基因的发现开启了血液肿瘤分子诊断的新篇章。随着分子生物学技术的发展，越来越多的融合基因被鉴定，并证实为AL的主要发病机制之一。目前2016年修订的WHO造血和淋巴组织肿瘤分类标准中明确列出的融合基因已经多达112种[5]–[6]。其中伴有RUNX1-RUNX1T1、PML-RARα、CBFβ-MYH11的患者，无需原始细胞比例>20％即可诊断为AML，因此融合基因检测是AML诊断分型的主要依据之一。此外，融合基因也是AML患者预后分层、微小残留病（MRD）监测和靶向治疗的主要分子标志物。

目前虽然国内外已有许多关于AML融合基因的研究，但是由于种族背景及样本量偏小使得不同研究结论存在一定差异。本研究对4192例初诊AML患者的52种融合基因结果进行系统性回顾分析，从而明确各种融合基因在AML患者中的分布情况，及不同融合基因家族患者的其他实验室检查特点。

## 病例与方法

1. 病例资料：收集2016年9月至2020年1月在天津见康华美医学实验室进行融合基因筛查，且确诊为AL的初诊患者6040例。其中AML患者4192例，ALL患者1777例，MPAL患者71例。AML患者中，292例（6.97％）为儿童，中位年龄8岁（1.8个月～14岁），男∶女为1.20∶1；3900例（93.03％）为成人，中位年龄55（15～91）岁，男∶女为1.22∶1。所有患者均根据2016年修订的WHO造血和淋巴组织肿瘤分类标准[5]–[6]，结合患者的病理形态学、流式细胞学、遗传学和分子生物学检查结果进行综合诊断。

2. 融合基因筛查：采用TRIzol法从患者的骨髓单个核细胞中提取总RNA，按照白血病融合基因检测试剂盒（厦门至善公司产品）说明书配置反应液，使用ABI7500扩增仪进行扩增反应。

52种融合基因包括BCR-ABL p210、BCR-ABL p190、PML-RARα S型、PML-RARα V型、PML-RARα L型、SIL-TAL1、E2A-HLF、TEL-AML1、MLL-AF4、E2A-PBX1、RUNX1-RUNX1T1、MLL-AF9、MLL-AF6、MLL-AF10、MLL-ELL、MLL-ENL、PLZF-RARα、STAT5b-RARα、NPM-MLF1、TEL-PDGFRB、FIP1L1-PDGFRA、AML1-MDS1/EVI1、AML1-MTG16、CBFβ-MYH11、DEK-CAN、TEL-ABL、ETV6-PDGFRA、NUP98-HOXA13、NUP98-HOXC11、NUP98-HOXD13、NUP98-HOXA9、NUP98-HOXA11、NUP98-PMX1、TEL-JAK2、MLL-AF17、MLL-AF1q、MLL-AF1p、MLL-AFX、MLL-SEPT6、NPM-RARα、FIP1L1-RARα、PRKAR1A-RARα、NUMA1-RARα、NPM-ALK、SET-CAN、TLS-ERG、EVI1、HOX11、HOX11L2、dup MLL、CALM-AF10、AML1-EAP。

3. 免疫表型分析：取患者骨髓液5 ml，肝素抗凝，经红细胞裂解液处理后，进行相关抗体标记，PBS洗涤。采用BC Navios多色流式细胞仪检测白血病相关抗原表达。

4. 基因突变筛查：通过Illumina NextSeq 550测序平台，对143个与血液肿瘤密切相关的基因外显子及旁侧内含子区域进行高通量测序，平均测序深度为1000×。用ANNOVAR对变异数据进行注释。利用genomeAD和1000Genomes人群数据库、COSMIC肿瘤数据库、Polyphen2和Mutationtaster功能数据库及实验室内部自建数据库对变异位点进行分析过滤，筛选出与疾病密切相关的变异位点。

5. 统计学处理：使用SPSS 25.0软件进行统计学分析。差异性分析采用*χ*^2^检验或Fisher确切概率法，*P*<0.05为差异有统计学意义。

## 结果

一、初诊AL患者融合基因表达情况

6040例初诊AL患者，综合病理形态学、流式细胞学、遗传学和分子生物学检测结果，最终确诊AML患者4192例（69.40％），ALL患者1777例（29.42％），MPAL患者71例（1.18％）。AL患者的融合基因阳性率为45.88％（2771/6040），AML、ALL及MPAL患者阳性率分别为46.47％（1948/4192）、45.24％（804/1777）、26.76％（19/71）。

二、AML患者融合基因分布特征

如表1所示，在1948例融合基因阳性AML患者中共检测到29种不同的融合基因，融合基因的表达呈现“幂律分布”，阳性率>1％的融合基因包括三种：PML-RARα（18.96％）、RUNX1-RUNX1T1（14.98％）、CBFβ-MYH11（5.46％）；阳性率<1％的融合基因占89.66％（26/29）。另外，检测到6例成人患者同时携带两种融合基因，占所有AML患者的0.14％，占融合基因阳性患者的0.30％。其中3例为CBFβ-MYH11和BCR-ABL双阳性，其余3例分别为PML-RARα和BCR-ABL、DEK-CAN和SET-CAN、TEL-ABL和MLL-ELL双阳性。

**表1:** 4192例初诊急性髓系白血病患者融合基因的分布情况

融合基因	例数（％）	性别（例）		年龄［例（％）］		
		男性（2304例）	女性（1888例）	0～14岁（292例）	>14岁（3900例）	*P*值
PML-RARα	795（18.96）	418	377	70（23.97）	725（18.60）	0.023
RUNX1-RUNX1T1	628（14.98）	385	243	76（26.03）	552（14.15）	<0.001
CBFβ-MYH11	229（5.46）	140	89	18（6.16）	211（5.41）	0.584
MLL-AF9	66（1.57）	31	35	11（3.77）	55（1.41）	0.004
MLL-AF6	37（0.88）	16	21	4（1.37）	33（0.85）	0.549
BCR-ABL	19（0.45）	12	7	1（0.34）	18（0.46）	0.873
MLL-ELL	31（0.74）	16	15	1（0.34）	30（0.77）	0.641
DEK-CAN	25（0.60）	12	13	2（0.68）	23（0.59）	0.849
MLL-AF10	30（0.72）	12	18	11（3.77）	19（0.49）	<0.001
NUP98-HOXA9	18（0.43）	10	8	0（0）	18（0.46）	1.000
TLS-ERG	18（0.43）	9	9	1（0.34）	17（0.44）	0.819
MLL-ENL	10（0.24）	5	5	1（0.34）	9（0.23）	0.515
SET-CAN	7（0.17）	6	1	2（0.68）	5（0.13）	0.080
MLL-AF1q	5（0.12）	0	5	3（1.03）	2（0.05）	0.003
AML1-MDS1/EVI1	4（0.10）	4	0	0（0）	4（0.10）	1.000
AML1-MTG16	4（0.10）	3	1	1（0.34）	3（0.08）	0.251
STAT5b-RARα	3（0.07）	2	1	0（0）	3（0.08）	1.000
MLL-AF17	3（0.07）	2	1	0（0）	3（0.08）	1.000
MLL-AF4	2（0.05）	1	1	0（0）	2（0.05）	1.000
NUP98-HOXD13	2（0.05）	2	0	0（0）	2（0.05）	1.000
PLZF-RARα	2（0.05）	1	1	0（0）	2（0.05）	1.000
NUP98-PMX1	1（0.02）	1	0	0（0）	1（0.03）	1.000
NPM-MLF1	1（0.02）	1	0	0（0）	1（0.03）	1.000
NUP98-HOXA13	1（0.02）	0	1	0（0）	1（0.03）	1.000
NUP98-HOXA11	1（0.02）	0	1	0（0）	1（0.03）	1.000
CBFβ-MYH11和BCR-ABL	3（0.07）	2	1	0（0）	3（0.08）	1.000
PML-RARα和BCR-ABL	1（0.02）	1	0	0（0）	1（0.03）	1.000
DEK-CAN和SET-CAN	1（0.02）	1	0	0（0）	1（0.03）	1.000
TEL-ABL和MLL-ELL	1（0.02）	0	1	0（0）	1（0.03）	1.000

69.18％（202/292）的儿童AML患者携带融合基因，而成人AML患者中融合基因阳性率为44.77％（1746/3900），二者差异具有统计学意义（*P*<0.001）。此外，PML-RARα、RUNX1-RUNX1T1、MLL-AF10和MLL-AF1q阳性患者在儿童AML中占比高于成人，而累及NUP98的融合基因阳性患者均为成人。

对所有AML患者按年龄段进行进一步分析，如图1所示，2～35岁患者的融合基因阳性率最高，约为70％，25岁以后阳性率随年龄增长逐渐降低，≥65岁的患者阳性率约为20％。2岁前PML-RARα和RUNX1-RUNX1T1的阳性率最低，随着年龄的增加，分别在26～35岁和9～14岁达到峰值，之后随着年龄的增长而下降。2岁前MLL相关融合基因（MLL-FG）和CBFβ-MYH11的阳性率最高，在幼儿和青春期逐渐下降，成年后均随年龄增长先增加后下降。

**图1:**
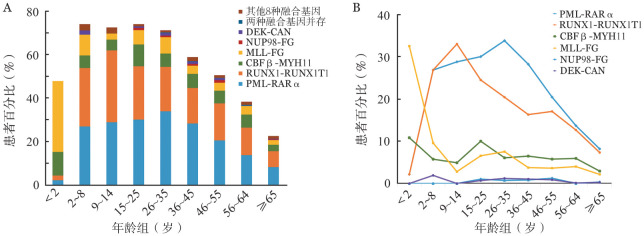
不同年龄组急性髓系白血病患者融合基因分布 A：各年龄组不同融合基因的分布特征；B：RUNX1-RUNX1T1、MLL相关融合基因（MLL-FG）、PML-RARα、CBFβ-MYH11、NUP98-FG和DEK-CAN阳性率随年龄增长的变化

三、不同融合基因家族的表达及各组患者的免疫表型和基因突变特征

我们分析了三类融合基因家族MLL-FG、RARα相关融合基因（RARα-FG）及NUP98相关融合基因（NUP98-FG）的表达及阳性患者免疫表型和基因突变特征。

1. MLL-FG的表达及阳性患者免疫表型特征：MLL-FG阳性患者共计184例（4.39％）。其中MLL-AF9阳性者最多（35.87％），其次为MLL-AF6（20.11％）、MLL-ELL（16.85％）、MLL-AF10（16.30％）和MLL-ENL（5.43％），MLL-AF1q、MLL-AF17、MLL-AF4的阳性率均<5％，未检测到MLL-AF1p、MLL-AFX和MLL-SEPT6。

如图2所示，将该组患者按年龄划分为儿童组及成人组。经统计学分析发现，儿童患者MLL-FG阳性率（31/202，15.35％）显著高于成人患者（153/1746，8.76％）；MLL-AF9在成人和儿童MLL-FG阳性患者中的比例相似；成人MLL-ELL的比例明显高于儿童患者（19.61％对3.23％，*P*＝0.026）；成人MLL-AF10和MLL-AF1q的比例较儿童患者低（12.42％对35.48％，*P*＝0.002；1.31％对9.68％，*P*＝0.034）；儿童患者未检测到MLL-AF17及MLL-AF4。

**图2:**
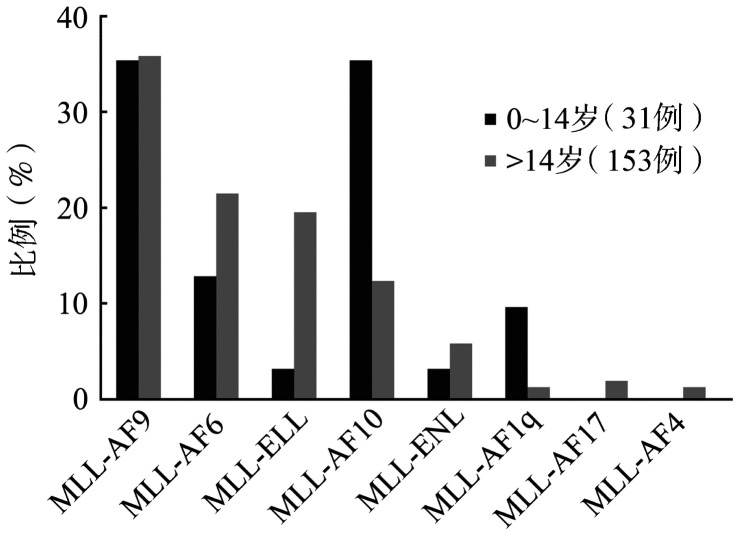
MLL相关融合基因在成人及儿童急性髓系白血病患者中的分布

分析147例MLL-FG阳性患者的免疫表型特征，结果显示，大多数患者表达髓系抗原（CD33、CD117、CD13、HLA-DR）及单核系抗原（CD64、CD4）；儿童AML-AF9患者均不表达CD34，但均表达HLA-DR，二者均为造血干/祖细胞表面标志；其他抗原表达差异无统计学意义（表2）。

**表2:** MLL相关融合基因阳性急性髓系白血病患者免疫表型特点

融合基因	例数	中位年龄（岁）	性别（例，男/女）	免疫表型［例（％）］									
				CD33	CD117	CD13	HLA-DR	CD34	CD64	CD4	CD11b	CD14	CD56
成人													
MLL-AF9	42	54	22/20	42（100.0）	29（69.0）	38（90.5）	33（78.6）	11（26.2）	38（90.5）	27（64.3）	23（54.8）	7（16.7）	18（42.9）
MLL-AF6	30	39	14/16	30（100.0）	29（96.7）	27（90.0）	30（100.0）	27（90.0）	27（90.0）	22（73.3）	15（50.0）	9（30.0）	6（20.0）
MLL-ELL	19	53	8/11	19（100.0）	16（84.2）	19（100.0）	14（73.7）	14（73.7）	13（68.4）	4（21.1）	4（21.1）	4（21.1）	0
MLL-AF10	13	47	5/8	13（100.0）	12（92.3）	7（53.8）	12（92.3）	6（46.2）	12（92.3）	11（84.6）	4（30.8）	1（7.7）	7（53.8）
MLL-ENL	9	48	4/5	9（100.0）	7（77.8）	8（88.9）	6（66.7）	2（22.2）	9（100.0）	7（77.8）	4（44.4）	2（22.2）	7（77.8）
MLL-AF1q	2	50.5	0/2	2（100.0）	1（50.0）	2（100.0）	2（100.0）	0（0）	2（100.0）	2（100.0）	1（50.0）	0（0）	0（0）
MLL-AF17	1	28	0/1	1（100.0）	1（100.0）	1（100.0）	1（100.0）	0（0）	1（100.0）	1（100.0）	0（0）	0（0）	0（0）
MLL-AF4	1	63	1/0	1（100.0）	0（0）	0（0）	1（100.0）	0（0）	1（100.0）	1（100.0）	1（100.0）	0（0）	0（0）
儿童													
MLL-AF9	11	1	6/5	11（100.0）	6（54.5）	5（45.5）	11（100.0）	0（0）	9（81.8）	9（81.8）	7（63.6）	4（36.4）	4（36.4）
MLL-AF6	4	10	0/4	4（100.0）	1（25.0）	4（100.0）	4（100.0）	1（25.0）	4（100.0）	4（100.0）	3（75.0）	1（25.0）	0（0）
MLL-ELL	1	5	1/0	1（100.0）	1（100.0）	1（100.0）	0（0）	1（100.0）	1（100.0）	0（0）	0（0）	0（0）	0（0）
MLL-AF10	11	1	5/6	11（100.0）	7（63.6）	8（72.7）	10（90.0）	5（45.5）	10（90.0）	10（90.0）	7（63.6）	0（0）	6（54.5）
MLL-ENL	1	5	1/0	1（100.0）	1（100.0）	1（100.0）	1（100.0）	1（100.0）	1（100.0）	1（100.0）	1（100.0）	0（0）	1（100.0）
MLL-AF1q	2	4	0/2	2（100.0）	1（50.0）	1（50.0）	2（100.0）	1（50.0）	2（100.0）	1（50.0）	1（50.0）	1（50.0）	1（50.0）

对34例患者进行基因突变分析，如图3所示，23例（67.6％）患者共发生10个基因的突变，按其功能可划分为6类。最常见的突变是RAS信号通路相关基因（52.9％，18/34），其次为酪氨酸激酶受体相关基因（17.6％，6/34）。RAS信号通路相关基因突变中，KRAS（60.0％，15/25）突变最多见。

**图3:**
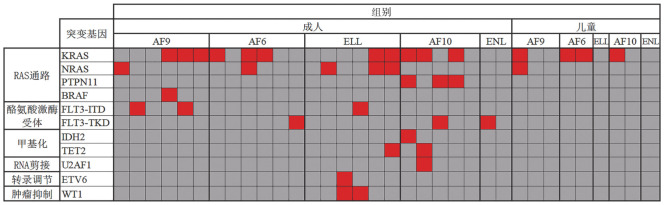
34例伴MLL相关融合基因（MLL-FG）急性髓系白血病患者基因突变情况 MLL-FG组25例成人和9例儿童患者突变检测结果。每列代表一例患者，每行代表一个基因。红色代表突变；深灰色代表野生型

2. NUP98-FG的表达及阳性患者免疫表型和基因突变特征：NUP98-FG阳性患者共计23例（0.55％），其中20例为伴t（7;11）（p15;p15）患者（伙伴基因为HOXA9、HOXA11和HOXA13），2例为NUP98-HOXD13，1例为NUP98-PMX1，未检测到NUP98-HOXC11阳性患者。

如表3所示，所有进行免疫表型分析的21例患者均表达CD34、CD33、CD117和CD13，19例患者表达HLA-DR。除3例伴t（7;11）（p15;p15）患者表达CD56，1例伴NUP98-HOXD13患者表达CD19外，无其他淋系相关抗原表达。

**表3:** NUP98相关融合基因阳性急性髓系白血病患者免疫表型和基因突变特点

例号	年龄（岁）	性别	伙伴基因	免疫表型							基因突变	
				CD34	CD33	CD117	CD13	HLA-DR	CD56	CD19	Ⅰ类突变	Ⅱ类突变
1	24	男	HOXA9	+	+	+	+	+	+	–	N/A	N/A
2	65	男	HOXA9	+	+	+	+	+	–	–	N/A	N/A
3	78	男	HOXA9	+	+	+	+	–	–	–	N/A	N/A
4	47	男	HOXA9	+	+	+	dim	+	–	–	N/A	N/A
5	52	男	HOXA9	+	+	+	+	+	+	–	NRAS	WT1
6	70	女	HOXA9	+	+	+	+	+	–	–	NRAS	TP53
7	29	女	HOXA9	+	+	+	+	+	–	–	N/A	N/A
8	32	女	HOXA9	+	+	+	+	+	–	–	N/A	N/A
9	39	女	HOXA9	+	+	+	+	+	–	–	N/A	N/A
10	66	男	HOXA9	+	+	+	+	dim	–	–	/	DNMT3A
11	32	男	HOXA9	+	+	+	+	+	–	–	FLT3-ITD	/
12	51	女	HOXA9	N/A	N/A	N/A	N/A	N/A	N/A	N/A	FLT3-ITD, NRAS, KRAS	/
13	55	男	HOXA9	+	+	+	+	+	–	–	FLT3-TKD	/
14	49	女	HOXA9	dim	+	+	+	+	–	–	IDH2, NRAS, KRAS	/
15	22	女	HOXA9	+	+	+	+	–	–	–	NRAS	WT1
16	46	女	HOXA9	N/A	N/A	N/A	N/A	N/A	N/A	N/A	N/A	N/A
17	48	男	HOXA9	+	+	+	+	dim	–	–	N/A	N/A
18	37	男	HOXA9	+	+	+	dim	+	–	–	N/A	N/A
19	47	女	HOXA11	dim	+	+	+	+	–	–	FLT3-ITD, NRAS	/
20	23	女	HOXA13	dim	+	+	+	dim	+	–	/	/
21	46	男	HOXD13	+	+	+	+	+	–	+	N/A	N/A
22	44	男	HOXD13	+	+	+	+	dim	–	–	FLT3-ITD	/
23	55	男	PMX1	+	+	+	+	dim	–	–	FLT3-ITD	/

注：N/A：未进行相关检测；/：未检测到该类突变；dim：弱表达

12例NUP98-FG阳性患者的基因突变筛查显示，11例（91.2％）患者共计发生7个基因突变。按其功能分为功能获得性突变（Ⅰ类突变）和功能缺失性突变（Ⅱ类突变）。7例患者仅携带Ⅰ类突变，1例仅携带Ⅱ类突变（DNMT3A），3例同时检测到Ⅰ类和Ⅱ类突变。Ⅰ类突变中以FLT3及NRAS突变为主，阳性率均为60.0％（6/10）。

3. RARα-FG的表达及阳性患者免疫表型特征：RARα-FG阳性患者共计800例（19.08％），其中PML-RARα阳性795例，STAT5b-RARα阳性3例，PLZF-RARα阳性2例；未见NPM-RARα、FIP1L1-RARα、PRKAR1A-RARα和NUMA1-RARα阳性患者。

对549例RARα-FG阳性患者的免疫表型进行分析（表4），CD34、HLA-DR表达较弱，MPO强表达。伴PML-RARα阳性AML患者与伴其他RARα-FG阳性患者的免疫表型特征差异无统计学意义。

**表4:** RARα相关融合基因阳性急性髓系白血病患者免疫表型特点

融合基因	例数	中位年龄（岁）	性别比（男∶女）	免疫表型［例（％）］							
				CD34	CD33	MPO	CD13	HLA-DR	CD64	CD56	CD9
STAT5b-RARα	2	30	1∶1	0	+：2（100）	+：2（100）	+：2（100）	0	dim：2（100）	+：2（100）	+：1（50）dim：1（50）
PLZF-RARα	2	65	1∶1	0	+：2（100）	+：1（50） dim：1（50）	+：2（100）	dim：1（50）	dim：2（100）	0	+：2(100）
PML-RARα	545	43	1.1∶1	+：29（5）	+：541（99）	+：480（88） dim：65（12）	+：536（98） dim：9（2%）	+：4（1） dim：30（6）	+：60（11） dim：479（88）	+：18（3） dim：53（10）	+：526（97） dim：16（3）

注：dim：弱表达

## 讨论

融合基因是AL特异性的分子标志物之一，在AL的诊疗过程中扮演重要的角色。

本研究回顾性分析了我国4192例初诊AML患者的52种融合基因检测结果，融合基因阳性率为46.47％，显著高于外国学者的研究（46.47％对22.82％，46.47％对38.80％，*P*<0.001）[7]–[8]，而接近我国学者的报道（41.21％）[9]。与之类似，本研究及Estey等[10]的报道均显示PML-RARα和RUNX1-RUNX1T1为AML最常见的两种融合基因，但本研究中二者阳性率更高（18.96％对13％，*P*<0.001；14.98％对7％，*P*<0.001），与我国其他研究结果相似（分别为16.7％和15.1％）[11]。CBFβ-MYH11、MLL-FG、DEK-CAN、NUP98-FG等阳性率则与国内外其他学者报道基本一致[10]–[14]。另外，本研究中不同年龄段患者各融合基因阳性率与马来西亚学者的研究总体趋势一致[15]。以上结果提示AML患者融合基因的表达在不同人群中总体趋势一致，均表现出幂律现象，但阳性率依然存在一定的种族差异。

为了探索不同融合基因家族的融合基因表达特点，本研究重点分析了MLL-FG、NUP98-FG及RARα-FG。MLL基因的伙伴基因数量众多，至少报道过77种，其中常见的9种MLL-FG占90％以上[16]–[17]，均已包含在本研究中。NUP98伙伴基因目前也已发现30多种，可大致分为两类，同源盒基因和非同源盒基因家族，以NUP98-HOXA9最为常见；本研究涉及的伙伴基因均属于同源盒基因家族。而另一占比较大的融合基因家族RARα-FG中PML-RARα占98％，其他伙伴基因发现至少14种。由此可以看出这三类融合基因家族表达也存在幂律现象。聚焦于研究阳性率高的融合基因固然重要，但同样需要重视筛查阳性率较低的融合基因。例如，PLZF-RARα和STAT5B-RARα阳性患者均对全反式维甲酸（ATRA）不敏感[18]–[20]，需要考虑联合化疗和造血干细胞移植，基因漏检则会影响治疗。

进一步对三组融合基因家族的其他实验室检查结果进行分析，未发现本研究三组患者分化抗原表达有明显差异。此外，我们发现MLL-FG和NUP98-FG组患者伴随的突变基因均以FLT3及RAS信号通路相关基因为主[17],[21]–[25]。AML的发病遵循“二次打击”的模式，表现为单独一种基因的改变不足以导致疾病的发生，是多个基因参与的过程。这些基因改变可以分为功能获得性突变（Ⅰ类突变）和功能缺失性突变（Ⅱ类突变）。Ⅰ类基因改变使细胞获得增殖和存活的优势，Ⅱ类基因改变使细胞分化受阻，两类基因突变共同作用导致白血病的发生，并在疾病进展中发挥作用[26]。目前关于AML的研究中，FLT3突变和NRAS相关信号通路基因突变基本上为预后不良的因素[27]–[28]，且目前已有相关的靶向药物应用于临床或处于试验阶段。因此，对伴随基因的研究有助于判断疾病的进展及寻找新的治疗靶点。

此外值得注意的是，本研究检测到6例成人患者同时携带两种融合基因。对这些基因进行功能分类，我们发现BCR-ABL、TEL-ABL和DEK-CAN融合基因属于功能获得性改变，而CBFβ-MYH11、PML-RARα和MLL-ELL属于功能缺失性改变。本研究中6例患者携带的两种融合基因均分别为功能获得性和功能缺失性，这也进一步印证了“二次打击”的模式。

综上，分析比较AML患者融合基因的分布特点及伴随基因的改变，可以了解儿童和成人患者的差异，了解不同重现性遗传学异常AML的伴随分子特点，有助于精准的诊断分型及预后判断，为临床制定治疗方案和策略提供依据。
